# From psychological wellbeing to distress: the role of psychological counseling interventions in university students

**DOI:** 10.3389/fpsyg.2025.1602009

**Published:** 2025-08-08

**Authors:** Francesco Craig, Gianluca Mariano Colella, Flaviana Tenuta, Martina Mauti, Antonio Gravina, Maria Luigia Calomino, Roberta Plastina, Alberto Polito, Angela Costabile

**Affiliations:** ^1^Department of Cultures, Education and Society, University of Calabria, Cosenza, Italy; ^2^Psychological Counseling Services, University of Calabria, Cosenza, Italy

**Keywords:** psychological wellbeing, counseling interventions, dual continua model, university students, academic engagement, psychological distress

## Abstract

**Introduction:**

University students encounter specific psychosocial challenges contributing to increasing psychological distress. The dual continua model posits that psychological wellbeing and distress are separate yet interrelated aspects of mental health. This study examines the relationships between psychological wellbeing, academic engagement, and mental distress in students seeking support from a University Psychological Counseling Service and evaluates the effectiveness of psychological counseling interventions in improving wellbeing and reducing distress.

**Methods:**

A mixed-method approach was adopted, incorporating both cross-sectional and longitudinal analyses. A total of 246 students completed self-report measures such as the Ryff's Psychological Wellbeing Scale, the SInAPSi Academic Engagement Scale, and the Clinical Outcomes in Routine Evaluation. For the longitudinal component, 90 students were assessed before (T0) and after (T1) six counseling sessions. Multiple regression and mediation analyses explored predictors of distress, while paired *t*-tests evaluated intervention outcomes.

**Results:**

Self-Acceptance (*p* < 0.001) and Environmental Mastery (*p* = 0.037) dimensions significantly predicted lower psychological distress. Academic engagement did not mediate the relationship between wellbeing and distress. Post-intervention, psychological distress decreased significantly (*p* < 0.001), with 56.7% of students falling over the clinical distress cut-off at T1 compared to 84.4% at T0. The Autonomy (*p* = 0.03) and Self-Acceptance (*p* = 0.002) dimensions showed significant post-intervention improvements. The current study emphasizes that mental health is a dynamic, multidimensional construct, including both psychological distress and wellbeing. Universities should integrate positive psychology into curricula and expand psychological counseling services to provide proactive support, focusing on resilience, stress management, and emotional regulation.

## Introduction

The psychological wellbeing of university students has been a concern for many years worldwide (Campbell et al., 2022; Chaudhry et al., 2024; Duffy et al., 2019). The dual continua model of mental health assumes that psychological wellbeing and psychological distress are two related, yet distinct dimensions of a unique mental health continuum (Keyes, 2005). Both dimensions can coexist independently, allowing individuals to experience low distress alongside low wellbeing or, conversely, high distress alongside robust wellbeing (Iasiello and Van Agteren, 2020).

Since 1986, the World Health Organization (WHO) views “wellbeing” as more than the absence of illness (Keyes, 1998; Ryff, 1989), and the socio-ecological theory emphasizes the individual, social and environmental determinants of wellbeing (Reupert, 2017). Psychological wellbeing is often assessed through Ryff's Psychological Wellbeing Scale (PWBS; Ryff, 1989). Psychological wellbeing is a multidimensional concept stemming from positive psychology and encircling a positive state of health that enables individuals to excel across mental, physical, emotional, and social dimensions (Ryff and Singer, 2008; Niemiec, 2024). The six dimensions of Ryff's PWBS include Self-Acceptance, Positive Relationships, Autonomy, Environmental Mastery, Purpose in Life, and Personal Growth, reflecting a *eudaimonic* perspective focused on life perception rather than the *hedonic* focus on positive feelings (happiness, positive emotions, and life satisfaction). Clinical studies reported that higher PWBS scores are associated with lower levels of depression and stress, enhancing emotional regulation, and improving overall health outcomes, highlighting its restorative role in mental health (Klainin-Yobas et al., 2021; Lopes and Nihei, 2021).

On the other hand, psychological distress reflects negative states, such as anxiety, depression, or stress (Viertiö et al., 2021). It negatively impacts mental health and undermines psychological wellbeing, limiting an individual's ability to function, cope with challenges, and maintain a balanced, fulfilling life (Sharp and Theiler, 2018). Enrollment in university is a pivotal transition in a young person's life, marked by significant changes and high expectations. Challenges include adapting to various aspects of academic life, such as independent living, establishing new social networks, navigating diverse learning styles, and managing growing financial responsibilities, all of which can significantly affect overall mental wellbeing (Worsley et al., 2021; Teixeira et al., 2022). Recent epidemiological research indicates that university students frequently report symptoms of depression and anxiety, along with high levels of perceived psychological stress (Asif et al., 2020; Ochnik et al., 2021; Auerbach et al., 2018). For instance, a longitudinal cohort study conducted between 2013 and 2021, involving over 350,000 students, reported that more than 60% met the diagnostic criteria for at least one mental health disorder (Lipson et al., 2022). Similarly, a meta-analysis of 64 studies, which included 100,187 university students, estimated the prevalence of depressive symptoms at 33.6% and anxious symptoms at 39.0% (Li et al., 2022).

In recent years, there has been a steady increase in the number of students seeking help at University Psychological Counseling Services, and the concerns they bring have become increasingly severe (Salimi et al., 2023; Adachi et al., 2020). This growing need reflects both a reduction in the stigma surrounding mental health and an evolving recognition of the importance of wellbeing in academic environments (Priestley et al., 2022). Beyond addressing mental health concerns (Craig et al., 2023), University Psychological Counseling Services might play a key role in fostering positive dimensions, such as personal growth, resilience, and academic engagement. Regardless of the critical function of these services, there remains a notable lack of longitudinal research examining the interplay between risk factors (e.g., academic burnout, social isolation) or protective factors (e.g., resilience, self-efficacy, and social support) in this population (Broglia et al., 2021).

It has been suggested that academic engagement may serve as a risk or protective factor for psychological distress of university students (Vizoso et al., 2018). Academic engagement is recognized as a multidimensional construct including social, affective, and behavioral components (Fredricks, 2022). This concept plays a crucial role in shaping students' capacity to form meaningful relationships, remain committed to their academic journey, and integrate their university experience into the broader context of their lives. Strong connections with institutions, faculty, and peers are key drivers of academic success and persistence (Martinot et al., 2022). Fostering engagement not only reduces dissatisfaction and boredom, but also enhances motivation and academic performance, offering benefits for students facing psycho-social vulnerabilities (Freda et al., 2021). Thus, academic engagement is a crucial element in students' lives, affecting individual vulnerability and how one responds to environmental stressors. Moreover, sociodemographic variables such as socioeconomic status, gender, cultural background, and living arrangements further contribute to students' wellbeing by influencing their access to resources, support systems, and their ability to navigate academic and personal challenges potentially shaping their levels of psychological wellbeing (Fuentes et al., 2022).

Despite the growing recognition of the importance of wellbeing among university students, the literature review shows that research about students' mental health focuses mainly on negative aspects. Furthermore, to our knowledge, no studies investigated the effectiveness of psychological counseling interventions within the framework of the dual continua model of mental health, specifically examining academic factors among university students. Therefore, the hypotheses proposed in this study are grounded in the epistemological assumptions of the dual continua model of mental health (Keyes, 2005), which highlights the importance of assessing mental health not only by reducing distress or clinical symptoms, but also by promoting positive functioning and wellbeing outcomes. This model builds upon the eudaimonic approach to wellbeing proposed by Ryff (1989), which conceptualizes psychological wellbeing as a multidimensional construct. These dimensions offer a comprehensive understanding of mental health beyond symptom reduction and serve as the foundation for exploring the interplay between wellbeing and psychological distress.

**Figure 1:**
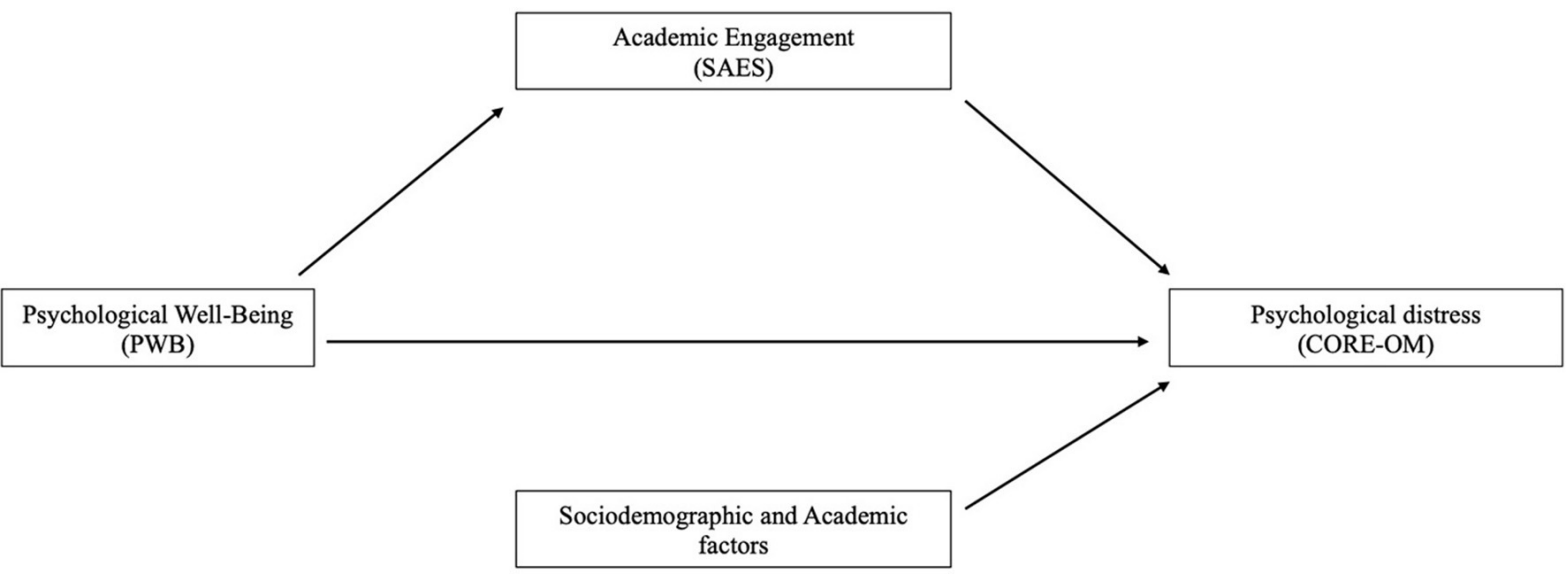
Conceptual model of the factors determining student's psychological distress.

Based on this theoretical framework, the first aim of this study is to explore the relationship between psychological wellbeing, academic engagement, and psychological distress in a broad sample of university students seeking help to the University Psychological Counseling Service. This framework is relevant for university settings, where the promotion of flourishing can impact students' ability to succeed academically and personally. Specifically, our pre-analyses conjectures are presented in Figure 1. We hypothesized that (a) psychological wellbeing would be positively related to academic engagement and negatively related psychological distress; (b) psychological wellbeing and academic engagement would influence psychological distress, while adjusting for the other confounders; and (c) academic engagement might mediate the relationship between psychological distress and psychological wellbeing.

Additionally, this study aims to assess the efficacy of counseling interventions in enhancing psychological wellbeing and alleviating distress among university students. Specifically, it will evaluate changes in these variables from baseline (T0) to post-intervention (T1), while examining the proportion of participants who meet clinical cut-off thresholds for psychological distress before and after the intervention.

## Methods

### Participants

All participants were recruited from university students who sought help at the psychological counseling service of our university. Inclusion criteria were age over 18 years, voluntary participation, expression of informed consent, ability to read and speak fluently in Italian, and absence of any self-reported disabilities that may hinder the completion of assessment tests. In addition, students were excluded if they were experiencing severe mental health conditions, such as hypomania or psychotic episodes, had recently undergone bereavement or a significant loss, or faced any other serious mental or physical health issues that could hinder their ability to participate in the program. Participants did not receive any reward.

The initial sample included 260 students. However, 14 participants were excluded as they did not complete the entire questionnaire. The final sample consisted of 246 students with a mean age of 22 years (SD = 3.55). Of these, 86 were male, 158 were female, and 2 did not respond. Regarding academic areas, 40% of students were enrolled in Education and Social programs, 25% in Medicine and Health programs, and 20% in Engineering and Technology disciplines. A total of 15% of participants did not specify their academic field of study. To evaluate the effectiveness of psychological counseling interventions, data were also collected from 90 students attending the university psychological counseling service and completed their sessions within the period from October 2024 to March 2025.

### Study design

The study protocol was approved by the Ethics Committee of the university (protocol number 0533627) and conducted in accordance with the principles outlined in the Declaration of Helsinki.

The research employed a mixed design incorporating both a cross-sectional and a longitudinal component. The cross-sectional phase explored key variables related to psychological wellbeing, academic engagement, and psychological distress in university students seeking support from the counseling service. Additionally, a longitudinal study was conducted to examine changes and trajectories in these variables over time. The assessment involves the administration of the identified measures at two key points: at baseline (T0) and post-intervention (T1, after 6 sessions).

The psychological assessment procedure was thoroughly explained to the students by qualified psychotherapists with over 3 years of expertise in providing psychological support. Before the assessment, the psychotherapists ensured that each student fully understood the purpose of the study, including data confidentiality and their right to withdraw at any time without any consequences. Detailed instructions on how to complete the assessment measures were given, addressing any concerns raised by the participants to ensure clarity and compliance. This approach aimed at creating a supportive environment, fostering trust and engagement throughout the process.

The longitudinal phase included questionnaire administration at T0, conducted immediately after a structured welcoming interview and before the start of clinical sessions within the counseling program. The follow-up assessment (T1) takes place upon program completion. The intervention consisted of six individual psychological counseling sessions, each lasting 50–60 min and led by five specialized psychotherapists. Sessions followed a structured format, beginning with an initial assessment to gather baseline data, identify concerns, and set goals. The core phase focused on evidence-based techniques tailored to individual needs, while the final session evaluated progress, explored long-term maintenance strategies, and administered post-intervention measures. Each interview was scheduled at weekly intervals.

### Measures

The participants completed a self-report questionnaire containing a set of different measures. *Sociodemographic information* was collected such as gender, age, degree course, and year of enrollment among the participants.

### Predictor variables

The Italian version of the *Psychological Wellbeing Scale* (PWBS; Ryff and Keyes, 1995; Italian validation by Ruini et al., 2003) was adopted to assess psychological wellbeing. The PWBS is a 42-item self-report scale assessing six areas of psychological wellbeing: Autonomy (7 items), Environmental Mastery (7 items), Personal Growth (7 items), Positive Relations with Others (7 items), Purpose in Life (7 items), and Self-Acceptance (7 items), on a 6-point Likert scale ranging from “*strongly disagree*” to “*strongly agree*.” The total PWB score can be calculated by adding together the scores of the six dimensions, ranging between 42 and 252. Higher scores indicate greater wellbeing in each domain. The Italian version of the scale showed robust psychometric properties across various age groups (Ruini et al., 2009). In our study, the total PWB scale showed acceptable reliability, with a Cronbach's α coefficient of 0.71.

The *SInAPSi Academic Engagement Scale* (SAES; Freda et al., 2021) was adopted to measure Academic Engagement. The SAES operationalizes engagement for university students with 29 items (on a 5-point Likert scale, ranging from “*not at all*” to “*totally*”) organized into 6 scales, corresponding to each of the six dimensions of the model: (1) Perception of the capability to persist in the university choice (e.g., I'd leave the university right away if I had an alternative); (2) University value and sense of belonging (e.g., Attending university is a great opportunity for me); (3) Value of university course (e.g., I find my studies very significant for my professional plans); (4) Relationships between university and relational network (e.g., I talk about my professional plans with my family); (5) Engagement with university peers (e.g., Studying with other students is useful to me); (6) Engagement with university professors (e.g., My instructors are interested in my opinions and what I say). The SAES has a valid factor structure and shows good convergent, discriminant, construct-related, and criterion-related validity (Freda et al., 2021). In our study, the (total) SAES scale demonstrated very good reliability, with a Cronbach' s α coefficient of 0.912.

### Outcome variable

The *Clinical Outcomes in Routine Evaluation—Outcome Measure* (CORE-OM) (Barkham et al., 2006; Evans et al., 2002; Italian validation by Palmieri et al., 2009) is a 34-item self-report questionnaire. It is a reliable instrument in clinical settings and a recommended outcome measure in Italian psychotherapy services for implementing routine evaluation. The CORE-OM investigates four domains: Wellbeing deficits (feelings about self and optimism about the future) (4 items), Problems/symptoms (depression, anxiety, physical problems, trauma) (12 items), Life Functioning difficulties (general day-to-day functioning, close relationships, social relationships) (12 items), Risk/harm (risk to self, risk to others) (6 items). Also, the CORE-OM provides total scores (all items (34 items). All items are scored on a 5-points Likert scale ranging from 0 (“*anchored all or most of the time*”) to 4 (“*sometimes*”), related to the previous week. The reliability of the Italian version of CORE–OM showed high internal consistency (α = 0.92) (Palmieri et al., 2009). In our study, the overall CORE-OM scale demonstrated very good reliability, with a Cronbach' s α coefficient of 0.916.

### Statistical methods

Descriptive statistics, tests for univariate normality (skewness and kurtosis), and bivariate correlation analysis (Pearson r) were performed to assess the distributions of variables and their interrelationships (Supplementary Table S1). Then, a multiple regression analysis was conducted to examine the predictors of overall psychological distress among the entire sample. To quantify the effects on psychological distress, we conducted a multiple regression analysis using the total CORE-OM score as the dependent variable, the six dimensions of PWBS as independent variables, and the six sub-dimensions of Academic Engagement as mediators. In addition, we included covariates such as gender, age, degree program, and year of enrollment to control for potential confounding factors.

Furthermore, to assess whether the effects of psychological wellbeing on the CORE-OM total score were partially meditated by academic engagement, we conducted a mediation analysis using the PROCESS macro (Model 4; Hayes, 2012). This analysis examined the direct and indirect effects of significant predictors on total psychological distress, with the six dimensions of academic engagement included as potential mediators.

Independent samples *t*-tests were conducted to explore potential sex-based differences in psychological wellbeing, distress, and academic engagement. A paired samples *t*-test was conducted to examine the differences in participants who completed the psychological counseling intervention. The aim was to assess the effectiveness of the intervention in improving the six dimensions of PWB scale and alleviating the CORE-OM subscale among university students. Additionally, a chi-square test was conducted, and the distributions of the CORE-OM cut-off scores were examined to determine whether a portion of the sample fell below the clinical cut-off for psychological distress following the intervention.

The statistically significant *p*-value was set at 0.05. All statistical analyses were conducted in SPSS software, version 23 (IBM SPSS Statistics for Windows, Version 23.0. Armonk, NY: IBM).

## Results

In Table 1, the sociodemographic data and the mean scores for the predictor and outcome variables are presented.

**Table 1:** Sociodemographic data and the mean scores for the predictor and outcome variables.

**Variable**	***n***	***%***
**Sex**		
Females	158	64.2
Males	86	35
NR	2	0.8
**Year of enrollment**		
1	51	20.7
2	61	24.8
3	57	23.2
4	37	15
5	25	10.2
Out of course	15	6.1
**Course of study**		
Bachelor's degree	143	58.1
Master's degree	78	31.7
Five-year degree	25	10.2
**CORE-OM**		
<cut-off	32	13
>cut-off	214	87
	Mean	SD
Age	22.59	3.55
**CORE-OM**		
Well-being deficits	2.40	0.8
Problems/symptoms	2.15	0.8
Life functioning difficulties	1.77	0.6
Risk/harm	0.28	0.47
Total	1.72	0.58
**SAES**		
Perception of the capability to persist in the university choice	3.85	0.93
University value and sense of belonging	4.1	0.68
Value of university course	3.94	0.88
Relationships between university and relational network	2.99	1.07
Engagement with university peers	3.65	0.93
Engagement with university professors	3.22	0.83
**PWBS (raw scores)**		
Autonomy	26.93	6.37
Environmental mastery	25.11	4.09
Personal growth	31.17	5.48
Positive relations with others	28.94	6.48
Purpose in life	30.3	6.21
Self-acceptance	23.13	6.75

CORE-OM, Clinical Outcomes in Routine Evaluation—Outcome Measure; SAES, SInAPSi Academic Engagement Scale; PWBS, Psychological Well-Being Scale; NR, No Response; SD, standard deviation.

A multiple regression analysis (Table 2) was conducted to examine the predictors of psychological distress. The Model indicated that 36% (*R*^2^ = 0.363, *p* < 0.001) of the variance in the CORE-OM-total score was accounted for by Environmental Mastery (*p* = 0.037) and Self-Acceptance (*p* < 0.001). Both variables were negatively associated with psychological distress, with lower levels of Environmental Mastery and Self-Acceptance leading to higher levels of psychological distress.

**Table 2:** Results for multiple regression of PWBS dimensions as predictors on the CORE-OM total score.

**Variable**	***t***	**95.0% confidence interval for B**		***p*-value**
		**Lower bound**	**Upper bound**	
Eta	0.684	−0.013	0.026	0.495
Sex	−1.389	−0.226	0.039	0.166
Course of study	−1.82	−0.187	0.007	0.07
Year of enrollment	−1.442	−0.082	0.013	0.151
**SAES**				
Perception of the capability to persist in the university choice	−1.032	−0.113	0.035	0.303
University value and sense of belonging	1.62	−0.029	0.301	0.107
Value of university course	−1.351	−0.2	0.037	0.178
Relationships between university and relational network	0.539	−0.053	0.092	0.590
Engagement with university peers	0.581	−0.062	0.113	0.562
Engagement with university professors	−0.912	−0.133	0.049	0.363
**PWBS**				
Autonomy	0.009	−0.012	0.012	0.993
Environmental mastery	−2.102	−0.044	−0.001	0.037^*^
Personal growth	−0.859	−0.023	0.009	0.391
Positive relations with others	−0.749	−0.017	0.008	0.455
Purpose in life	−0.244	−0.016	0.012	0.808
Self-acceptance	−5.021	−0.049	−0.021	0.000^*^

CORE-OM, Clinical Outcomes in Routine Evaluation—Outcome Measure; SAES, SInAPSi Academic Engagement Scale; PWBS, Psychological Well-Being Scale. ^*^p < 0.005.

**Figure 2:**
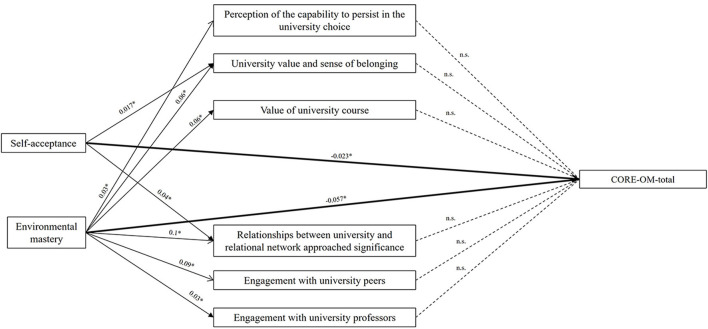
Mediation analysis results of the factors determining student psychological wellbeing. ^*^p < 0.005.

Furthermore, a mediation analysis (Figure 2, Table 3) was conducted through the PROCESS macro (Model 4) to examine the direct and indirect effects of Self-Acceptance and Environmental Mastery on total psychological distress, with six dimensions of academic engagement included as potential mediators. The analyses revealed a significant direct effect of Self-Acceptance on psychological distress (CORE-OM-total), with higher levels associated with lower psychological distress (β = −0.0237, *p* < 0.001). The model explained 14.92% of the variance in CORE-OM-total score (*R*^2^ = 0.1492, *p* < 0.001). Regarding the mediating variables, none of the engagement dimensions significantly mediated the relationship between Self-Acceptance and CORE-OM-total score. The total indirect effect was not significant. Similarly, none of the specific indirect effects through the engagement dimensions reached statistical significance. Self-Acceptance showed significant direct associations with the engagement dimensions such as Perception of the capability to persist in the university choice (*p* = 0.037), University value and sense of belonging (*p* < 0.0001), Value of university course (*p* < 0.0001), Relationships between university and relational network (*p* < 0.0001), Engagement with university peers (*p* < 0.0001), and with university professors (*p* = 0.0004); indicating that higher Self-Acceptance was positively linked to these aspects of engagement.

**Table 3:** Mediation and direct or indirect effects of PWBS and Academic Engagement dimensions on CORE-OM total scores.

**Pathway**	**Coefficient (β)**	**Standard error (se)**	***t*-value**	***p*-value**	**LLCI**	**ULCI**
Self-acceptance → CORE-OM-total	−0.0237	0.0056	−4.238	0.000^*^	−0.034	−0.012
Total indirect effect	−0.003	0.0024	−1.242	0.214	−0.007	0.0017
Self-acceptance → perception of the capability to persist in the university choice → CORE-OM-total	−0.0003	0.0006	−0.501	0.6179	−0.001	0.0009
Self-acceptance → university value and sense of belonging → CORE-OM-total	0.0019	0.0019	1.0038	0.3152	−0.001	0.0062
Self-acceptance → value of university course → CORE-OM-total	−0.0016	0.0015	−1.063	0.2883	−0.005	0.0008
Self-acceptance → relationships between university and relational network → CORE-OM-total	−0.0015	0.0017	−0.884	0.3772	−0.004	0.0018
Self-acceptance → engagement with university peers → CORE-OM-total	−0.0011	0.0011	−0.990	0.3225	−0.003	0.0006
Self-acceptance → engagement with university professors → CORE-OM-total	−0.0003	0.0007	−0.448	0.6532	−0.002	0.0009
Environmental mastery → CORE-OM-total	−0.0579	0.0093	−62.14	0.000^*^	−0.076	−0.039
Total indirect effect	−0.0034	0.0053	−0.641	0.521	−0.014	0.0064
Environmental mastery → perception of the capability to persist in the university choice	−0.0016	0.0014	−11.41	0.253	−0.004	0.0008
Environmental mastery → university value and sense of belonging	0.0098	0.0058	16.923	0.091	−0.001	0.0214
Environmental mastery → value of university course	−0.0071	0.0043	−16.51	0.099	−0.016	0.0008
Environmental mastery → relationships between university and relational network	−0.0019	0.0038	−0.497	0.619	−0.009	0.0053
Environmental mastery → engagement with university peers	−0.0009	0.0046	−0.192	0.847	−0.009	0.0078
Environmental mastery → engagement with university professors	−0.0018	0.0019	−0.947	0.343	−0.006	0.0017

PWBS, Psychological Well-Being Scale; CORE-OM, Clinical Outcomes in Routine Evaluation—Outcome Measure. ^*^p < 0.005.

Female students reported significantly higher scores than males (Supplementary Table S2) in the Wellbeing Deficits subscale of the CORE-OM (*p* = 0.003). Regarding academic engagement (SAES), females scored significantly higher in the Perception of the capability to persist in the university choice (*p* = 0.002), University value and sense of belonging (*p* = 0.008), and Relationships between university and relational network (*p* = 0.022).

The CORE-OM-total score was significantly influenced by Environmental Mastery (*p* < 0.0001) score, showing a negative direct effect. The Environmental Mastery showed significant direct associations with all engagement dimensions: Perception of the capability to persist in the university choice (*p* = 0.02), University value and sense of belonging (*p* < 0.0001), Value of university course (*p* < 0.0001), Relationships between university and relational network (*p* < 0.0001), Engagement with university peers (*p* < 0.0001), and with university professors (*p* = 0.0004). However, indirect effects through the six engagement measures were not significant.

Regarding longitudinal data, at T0, most participants (84.4%) were classified as students who exceeded the clinical cutoff of CORE-OM-total, while at T1, 56.7% exceeded the cutoff (Table 4). A chi-square test revealed significant differences between students exceeding the cutoff at T0 vs. T1 (*p* < 0.001). Overall, the percentage of students who did not exceeded the cutoff increased from 15.6% at T0 to 41.9% at T1, while the percentage of students who exceeded the cutoff decreased from 83.9% at T0 to 43.3% at T1. This change indicates a shift in the classification of participants over time, suggesting a significant improvement in their clinical status following the psychological counseling sessions.

**Table 4:** Differences in the prevalence of students exceeding the CORE-OM cut-off at T0 and T1 (observed vs. expected values).

**Group**	** <Cut-off**	**>Cut-off**	**Value**	***p*-value**
CORE-OM			10.021	0.003^*^
T0				
Count	10	52		
Expected count	18	44		
% within group	16.1%	83.9%		
T1				
Count	26	36		
Expected count	18	44		
% Within group	41.9%	58.1%		

CORE-OM, Clinical Outcomes in Routine Evaluation—Outcome Measure. ^*^p < 0.005.

The paired samples *t*-test revealed significant differences across multiple measures assessed at the two distinct time points (Table 5). The Wellbeing Deficits scale showed a significant decrease from the first to the second assessment (*p* = 0.001). Similarly, the Problems/Symptoms scale demonstrated a significant reduction (*p* = 0.005). Life Functioning Difficulties also significantly declined (*p* < 0.001). Moreover, overall psychological distress (CORE-OM total) exhibited a significant decrease over time (*p* < 0.001).

**Table 5:** Paired *t*-tests results for CORE-OM, SAES and PWBS scores in university students attending the University Psychological Counseling Services.

**Variable**	**T0 Mean ±SD**	**T1 Mean ±SD**	***t***	***p*-value**
**CORE-OM**				
Wellbeing deficits	2.38 ± 0.77	1.71 ± 0.88	5.31	0.001^*^
Problems/symptoms	2.10 ± 0.77	1.45 ± 0.72	2.89	<0.005^*^
Life functioning difficulties	1.77 ± 0.67	1.31 ± 0.59	5.36	<0.001^*^
Risk/harm	0.25 ± 0.39	0.15 ± 0.35	1.91	0.59^*^
Total	1.68 ± 0.6	1.20 ± 0.56	5.67	<0.001^*^
**SAES**				
Perception of the capability to persist in the university choice	3.99 ± 0.91	3.99 ± 0.97	0.025	0.98
University value and sense of belonging	4.25 ± 0.58	4.2 ± 0.7	0.457	0.65
Value of university course	4.08 ± 0.78	4.01 ± 0.79	0.506	0.615
Relationships between university and relational network	3.17 ± 1.03	3.22 ± 1.1	−0.244	0.808
Engagement with university peers	3.52 ± 0.9	3.62 ± 1.04	−0.578	0.565
Engagement with university professors	3.15 ± 0.91	3.30 ± 0.9	−1.172	0.246
**PWBS (raw scores)**				
Autonomy	26.72 ± 6.77	28.26 ± 6.18	−2.205	0.03^*^
Environmental mastery	25.38 ± 3.08	26.39 ± 4.59	−1.663	0.1
Personal growth	31.70 ± 5.15	33.09 ± 5.12	−1.961	0.53
Positive relations with others	28.23 ± 6.8	30.03 ± 6.98	−1.364	0.176
Purpose in life	30.23 ± 6.58	30.72 ± 6.64	−0.592	0.555
Self-acceptance	22.92 ± 6.64	25.91 ± 7.21	−3.239	0.002^*^

CORE-OM, Clinical Outcomes in Routine Evaluation—Outcome Measure; SAES, SInAPSi Academic Engagement Scale; PWBS, Psychological Wellbeing Scale; NR, No Response; SD, standard deviation. ^*^p < 0.005.

Regarding the PWBS, the Autonomy dimension showed a significant increase between the two time points (*p* = 0.003). Likewise, the Self-Acceptance dimension increased significantly (*p* = 0.002).

## Discussion

The main aim of this study was to examine the association between psychological wellbeing and distress in university students approaching the University Psychological Counseling Service. By doing so, our goal was to further validate the relevance of the dual continua model of mental health (Kraiss et al., 2023), assuming the discriminant validity of psychological distress and mental wellbeing (Hides et al., 2020). Then, cross-sectional and longitudinal data were collected, and analyses were conducted to examine the interplay between academic factors, as well as to assess the efficacy of counseling interventions in enhancing psychological wellbeing and reducing psychological distress among university students.

The results of our study highlight the importance of considering both wellbeing and distress when evaluating students' mental health, particularly in the context of University Psychological Counseling Services. We found that psychological wellbeing significantly predicted psychological distress, with Self-Acceptance and Environmental Mastery emerging as key protective factors. Specifically, these dimensions were negatively associated with psychopathological symptoms, suggesting that students with a lower sense of self-acceptance and perceived control over their surroundings are more vulnerable to psychological distress. This finding is in line with previous research indicating that low levels of Self-Acceptance, or the ability to recognize and embrace one's own strengths and limitations, can reduce emotional resilience and increase vulnerability to anxiety and depression disorders (Faustino et al., 2020; Trompetter et al., 2017; Zimmermann et al., 2021). Environmental Mastery, or the perception of being able to effectively manage one's surroundings, contributes to develop a sense of competence and stability, further mitigating psychological distress (Buratta et al., 2023). These two dimensions appear to protect from mental health difficulties, reinforcing the need for university-based interventions focusing on students' capacity for self-reflection, adaptive coping, and proactive problem-solving (Haliwa et al., 2022; Moeller et al., 2022). However, it is important to acknowledge that our sample consisted exclusively of students from a single university, which limits the generalizability of the findings. Future studies should involve larger, multisite samples to enhance external validity.

In the theoretical framework of the scientific discourse on mental health, these findings support the dual continua model which posits that mental health is not merely the absence of psychological distress, but also the presence of psychological wellbeing. Clinical studies provided substantial evidence in favor of this model, highlighting impairments in wellbeing among individuals with mood and anxiety disorders (Blasco-Belled et al., 2021; Franken et al., 2018). Khumalo et al. (2022) provided evidence for the model through factor and latent class analyses, reinforcing the idea that psychological wellbeing and distress are distinct yet interrelated dimensions of mental health. Other studies have suggested that wellbeing and distress should not be viewed as opposite poles of a single continuum, but rather as coexisting constructs that can independently fluctuate (Westerhof et al., 2023; Yeo and Suárez, 2022). Opposing perspectives argued that psychological wellbeing and distress represent entirely separate constructs, questioning the applicability of the dual continua model and suggesting that wellbeing should be conceptualized as the mere absence of distress (Kent et al., 2025; Lamers et al., 2011). In this vein, Kraiss et al. (2023) reported that psychological distress and wellbeing are only moderately associated when examined within individuals, suggesting that the dual continua model might not be applicable for everyone. Thus, although the debate remains open regarding the relationship between psychological distress and wellbeing, the current study underscores the importance of considering positive psychological functioning as an interrelated indicator of mental health in relation to psychological distress. This perspective carries significant theoretical implications. Specifically, higher levels of Self-Acceptance and Environmental Mastery were associated with lower psychological distress, pointing to a meaningful connection between the two constructs without suggesting they are interchangeable. Moreover, the absence of a mediating effect and the persistence of distress in some participants, despite gains in wellbeing, indicate that these dimensions can coexist and evolve independently. This pattern supports the view that mental health involves more than the mere absence of symptoms and includes the promotion of positive psychological resources.

Contrary to our expectations, we did not find a significant direct relationship between the sub-dimensions of academic engagement and psychological distress. Furthermore, academic engagement did not mediate the relationship between psychological wellbeing and distress. One possible explanation for this outcome is that psychological wellbeing contributes to a student's overall functioning, while academic engagement may be influenced by additional factors beyond wellbeing (Ma and Bennett, 2021; Sadoughi and Hejazi, 2023). Engagement in academic activities may not be sufficient to buffer against psychological distress, particularly if students are experiencing external stressors, academic burnout, or underlying mental health conditions (Ugwu et al., 2013). This finding highlights the complexity of the relationship between wellbeing, engagement, and distress (Keyes, 2005; Suldo and Shaffer, 2008). While previous research has emphasized the positive role of engagement in promoting academic success (Upadyaya and Salmela-Aro, 2013), its direct impact on mental health may be more nuanced, potentially requiring additional mediating or moderating variables, such as resilience, social support, or intrinsic motivation (Yu and Chae, 2020). Each of these variables has demonstrated a strong relationship with student wellbeing and engagement in empirical research. Resilience helps students thrive despite stress (Pidgeon et al., 2014), supportive social networks protect mental health under pressure (Breitenstein et al., 2025), and intrinsic motivation fosters both engagement and emotional wellbeing (Passeggia et al., 2023; Qureshi et al., 2024). Future studies should explore these factors as potential mediators or moderators in the complex dynamics between academic engagement, wellbeing and distress.

Although sex was included as a covariate in the regression analyses and did not emerge as a significant predictor, we conducted additional exploratory comparisons to assess potential sex-based differences. Results indicated that female students reported higher levels of wellbeing deficits and greater academic engagement, particularly in persistence, sense of belonging, and relational connectedness. These findings are consistent with previous research suggesting that female students tend to report greater emotional distress (Graves et al., 2021; Vuelvas-Olmos et al., 2023), while also displaying stronger academic motivation and social integration (Wang and Zhang, 2024). This dual pattern may reflect sex-based differences in emotional coping, with women more likely to internalize distress while simultaneously engaging with support networks and academic goals. No statistically significant differences were observed in the PWBS scores. However, some dimensions such as Personal Growth, Positive Relations, and Purpose in Life showed trends toward borderline significance in favor of female participants. These findings partially align with prior studies reporting sex-based differences in wellbeing profiles, with men typically scoring higher in Self-Acceptance and Autonomy, and women in Personal Growth and Positive Relations (Matud et al., 2019). Research should further investigate sex-related variations in psychological wellbeing using adequately powered designs, as these distinctions may have important implications for targeted intervention strategies in university settings.

Longitudinal analyses were conducted to assess pre-post changes following the counseling intervention. The findings showed a significant reduction in psychological distress among students following the counseling intervention, as evidenced by the decrease in those exceeding the CORE-OM clinical cut-off. This is an encouraging outcome, indicating that for many students, the psychological support provided by the University Psychological Counseling Service effectively alleviates distress and mitigates risk behaviors (Cerutti et al., 2022; Stewart et al., 2020; Murray et al., 2016). However, a considerable percentage of students remained within the at-risk category even after completing the brief counseling intervention (56.7%). This indicates that, while the brief counseling sessions were beneficial for many, they may not be sufficient for students experiencing more severe psychological challenges. These students likely require more intensive, individualized, or longer-term therapeutic support beyond the scope of the current University Psychological Counseling Service framework (Erekson et al., 2023; Strepparava et al., 2017). While short-term counseling services play a crucial role in providing immediate support, they may not be sufficient for individuals with more complex or enduring psychological difficulties (Bani et al., 2022).

Interestingly, we showed a significant improvement in psychological wellbeing, particularly in the dimensions of Self-Acceptance and Autonomy after counseling sessions. The significant increase in Self-Acceptance might suggest that, over the course of the counseling sessions, students developed a more positive perception of themselves. This aspect is crucial, as self-acceptance is a determining factor for overall psychological wellbeing and the ability to face daily challenges resiliently. Similarly, the significant increase in Autonomy highlights a greater ability of participants to self-determine and make independent decisions. This is a key element for optimal psychological functioning, as it allows individuals to pursue personal goals aligned with their values and desires, reducing dependence on external influences. These findings are consistent with previous studies showing that psychological counseling interventions significantly enhance wellbeing and decrease distress symptoms (Vescovelli et al., 2017). A recent meta-analysis (Van Dierendonck and Lam, 2023) examined the effectiveness of different types of interventions aimed at enhancing eudaimonic wellbeing across more than 70 studies. The meta-analytic results indicated that the most substantial effects were observed in the dimensions Environmental Mastery, Self-Acceptance and Personal Growth. Conversely, the weakest effects were found in the domains of Positive Relations with Others and Autonomy. While further research is needed to fully comprehend and apply the principles of positive psychology, our results indicate that it can serve a significant role in counseling interventions (Smith et al., 2021), particularly in university students (Prince, 2015). Indeed, goals of counseling can be comprised as both the restoration of wellbeing (remediating problems) and the promotion of wellbeing (growth and promotion). However, it will be crucial for University Psychological Counseling Services to integrate traditional interventions with various positive psychological approaches, including practicing forgiveness, participating in happiness training, keeping a gratitude journal, reflecting on positive experiences, engaging in meditation and mindfulness, writing a gratitude letter, performing acts of kindness, counting one's blessings, participating in productive activities, undergoing resilience training, nurturing relationships, and engaging in physical activities that promote wellbeing (Herbert, 2022). This integrative approach is further supported by recent evidence demonstrating that brief, structured interventions rooted in cognitive-behavioral therapy, positive psychology, or solution-focused models can meaningfully reduce psychological distress and enhance psychological resources in university student populations (Atik et al., 2023; Reschke et al., 2024; Wang et al., 2024; Barnett et al., 2021).

In this context, future research should advance along two key directions. First, it is important to explore individual trajectories of psychological change over time, using longitudinal modeling approaches such as growth curve or latent class analysis. Second, future studies should assess the effectiveness of evidence-based therapeutic approaches adapted to the university context. These could include Acceptance and Commitment Therapy (ACT), which enhances psychological flexibility (Levin et al., 2017); Mindfulness-Based Stress Reduction (MBSR), which improves emotional regulation (Sanilevici et al., 2021); and positive psychology interventions such as gratitude journaling and strengths identification (Datu et al., 2018). Psychoeducational programs focused on resilience have also shown encouraging results in reducing distress and fostering adaptive coping (Houston et al., 2017). Evaluating these interventions across diverse student populations and formats will be crucial to strengthen the impact and scalability of university counseling services.

## Limitations

Several limitations should be considered when interpreting these findings. Firstly, the sample size was limited and derived from a single university, which may affect the generalizability of the results and limit their representativeness of the broader student population. Furthermore, as the findings cannot be generalized to clinical samples, this may limit their applicability to samples in which the dual-continua model might manifest less distinctly (Peter et al., 2011).

Another limitation is the lack of sample clustering, which could have provided more nuanced insights into specific subgroups. A recent study in a large non-clinical adult population found that the PWBS network model identified four dimensions, with Self-Acceptance, Life Purpose, And Environmental Mastery clustering together, and Self-Acceptance emerging as the most central dimension (Blasco-Belled and Alsinet, 2022). Future studies should further explore the stability of the PWBS network model and its implications for mental health interventions

Furthermore, interventions utilized in the counseling sessions were non-specific, which may limit the ability to attribute improvements in psychological wellbeing to therapeutic techniques or approaches. Therefore, it is essential to develop and test effective intervention strategies that sustainably enhance psychological wellbeing, ensure a lasting impact, and can be widely implemented while also benefiting both physical and mental health.

## Conclusion

To the best of our knowledge, this is the first study to use both cross-sectional and longitudinal data, emphasizing the importance of applying a widely used framework to measure the dual-continua model of mental health in students seeking help from university counseling services. By applying this model, the study enhances our understanding of how mental health is not simply the absence of mental illness but rather a dynamic, multidimensional construct that include also psychological wellbeing dimensions. Specifically, the results emphasize the importance of measuring both psychological wellbeing and distress to gain a comprehensive understanding of university students' experiences with the current University Psychological Counseling Services. Secondly, our findings suggest that psychological counseling interventions are effective in enhancing wellbeing and reducing distress. Although a portion of students remained in the at-risk category after completing the sessions, the overall results are promising, demonstrating that psychological interventions have the potential to improve wellbeing dimension such as Self-Acceptance and Autonomy. The study indicates that various types of therapeutic interventions can contribute to wellbeing, with benefits arising from different formats, delivery methods, across non-clinical populations. However, further research focusing on clinical populations is needed to better understand the full scope of these interventions' impact. In conclusion, University Psychological Counseling Services play a pivotal role in fostering the psychological wellbeing of students, which in turn contributes to their academic success. To effectively support student wellbeing, universities should consider integrating positive psychology into their curricula, offering training programs that promote resilience, gratitude, and stress management.

## Data Availability

Publicly available datasets were analyzed in this study. This data can be found here: The raw data supporting the conclusions of this article will be made available by the authors.
