# Supplementary material for: From psychological wellbeing to distress: the role of psychological counseling interventions in university students
Source: Front Psychol. 2025 Aug 8;16:1602009. doi: 10.3389/fpsyg.2025.1602009 (PMC12370516; doi:10.3389/fpsyg.2025.1602009)
Supplement: Supplementary file 1 [file Data_Sheet_1.pdf]

Supplementary Material

TABLE S1. Correlational analysys between CORE-OM, SAES and PWBS dimensions

		1	2	3	4	5	6	7	8	9	10	11	12	13	14	15	16	17	18
1	Age	1	0.008	0.010	0.010	-0.039	0.004	0.012	0.026	-0.068	-0.059	-0.090	-0.040	0.071	0.059	0.066	0.077	-0.004	-0.028
2	Well-being deficits		1	0.742**	0.670**	0.345**	0.821**	-0.027	-0.168**	-0.216**	-0.124	-0.161*	-0.102	-0.269**	-0.362**	-0.279**	-0.184**	-0.287**	-0.510**
3	Problems/symptoms			1	0.694**	0.488**	0.934**	-0.120	-0.116	-0.164**	-0.119	-0.143*	-0.115	-0.251**	-0.361**	-0.317**	-0.243**	-0.221**	-0.466**
4	Life Functioning difficulties				1	0.454**	0.881**	-0.123	-0.223**	-0.250**	-0.262**	-0.302**	-0.195**	-0.298**	-0.442**	-0.397**	-0.405**	-0.341**	-0.562**
5	Risk/harm					1	0.605**	-0.139*	-0.118	-0.095	-0.200**	-0.118	-0.050	-0.130*	-0.264**	-0.222**	-0.274**	-0.186**	-0.275**
6	Total						1	-0.128*	-0.183**	-0.221**	-0.203**	-0.224**	-0.151*	-0.295**	-0.435**	-0.378**	-0.337**	-0.306**	-0.557**
7	Perception of the capability to persist in the university choice							1	0.422**	0.345**	0.125*	0.153*	0.064	0.042	0.146*	0.186**	0.171**	0.158*	0.133*
8	University value and sense of belonging								1	0.764**	0.455**	0.488**	0.376**	0.160*	0.383**	0.300**	0.245**	0.480**	0.319**
9	Value of university course									1	0.352**	0.484**	0.341**	0.111	0.308**	0.157*	0.217**	0.473**	0.304**

10	Relationships between university and relational network										1	0.400**	0.267**	0.240**	0.388**	0.308**	0.432**	0.423**	0.395**
11	Engagement with university peers											1	0.400**	0.106	0.399**	0.334**	0.378**	0.337**	0.342**
12	Engagement with university professors												1	0.055	0.180**	0.165**	0.276**	0.052	0.224**
13	autonomy													1	0.381**	0.448**	0.235**	0.334**	0.489**
14	environmental mastery														1	0.491**	0.466**	0.575**	0.599**
15	personal growth															1	0.460**	0.455**	0.593**
16	positive relations with others																1	0.342**	0.481**
17	purpose in life																	1	0.461**
18	self-acceptance																		1
	Skewness	2,586	-0,206	-0,013	0,109	2,176	0,131	-0,693	-0,549	-0,869	0,112	-0,565	0,011	-0,191	0,108	-0,173	-0,214	-0,512	-0,02
	Kurtosis	11,227	-0,648	-0,656	-0,494	4,641	-0,623	-0,318	-0,273	0,117	-0,801	-0,387	-0,125	-0,001	-0,342	-0,433	-0,2	-0,306	-0,385

Clinical Outcomes in Routine Evaluation – Outcome Measure (CORE-OM); SInAPSi Academic Engagement Scale (SAES); Psychological Well-Being Scale (PWBS);**p<0.01; *p< 0.05 level

TABLE S2. Sex-based comparisons of psychological distress, academic engagement, and psychological well-being

Variable	Female Mean±SD	Male Mean±SD	*p_value*
**CORE-OM**			
Well-being deficits	2.51 ± 0.78	2.19 ± 0.81	0.003*
Problems/symptoms	2.17 ± 0.83	2.1 ± 0.73	0.48
Life Functioning difficulties	1.75 ± 0.61	1.78 ± 0.58	0.781
Risk/harm	0.28 ± 0.44	0.29 ± 0.52	0.783
Total	1.73 ± 0.59	1.68 ± 0.54	0.496
**SAES**			
Perception of the capability to persist in the university choice	3.98 ± 0.9	3.6 ± 0.94	0.002*
University value and sense of belonging	4.19 ± 0.66	3.95 ± 0.68	0.008*
Value of university course	3.98 ± 0.89	3.88 ± 0.85	0.42
Relationships between university and relational network	3.11 ± 1.11	2.79 ± 0.95	0.02*
Engagement with university peers	3.65 ± 0.93	3.66 ± 0.92	0.931
Engagement with university professors	3.16 ± 0.85	3.33 ± 0.8	0.129
**PWBS (raw scores)**			
Autonomy	26.75 ± 6.51	27.28 ± 6.2	0.536
Environmental Mastery	25.53 ± 4.03	24.49 ± 4.1	0.058
Personal Growth	31.62 ± 5.58	30.42 ± 5.2	0.1
Positive Relations with Others	29.51 ± 6.43	28.01 ± 6.47	0.083
Purpose in Life	30.94 ± 5.98	29.33 ± 6.44	0.052
Self-Acceptance	23.28 ± 6.99	23.02 ± 6.28	0.778

Clinical Outcomes in Routine Evaluation – Outcome Measure (CORE-OM); SInAPSi Academic Engagement Scale (SAES); Psychological Well-Being Scale (PWBS); $p < 0.005$*
